# Air Leak Test: Extending the Horizon From the Bowel to Pancreatoduodenectomy

**DOI:** 10.7759/cureus.81172

**Published:** 2025-03-25

**Authors:** Ajay Sharma, Peeyush Varshney, Vinay Kumar Mahala, Maunil Tomar, Anand Nagar, Harshil Shaileshbhai Shah, Milind Akhani, Rajeev R Patil, Akshat Dhanuka, Rajendra Prasad Choubey

**Affiliations:** 1 Surgical Gastroenterology, Mahatma Gandhi Medical College and Hospital, Jaipur, Jaipur, IND; 2 Surgical Gastroenterology, All India Institute of Medical Sciences, Jodhpur, Jodhpur, IND; 3 Surgical Gastroenterology, Mahatma Gandhi Medical College and Research Institute, Jaipur, Jaipur, IND; 4 Surgical Gastroenterology, Mahatma Gandhi University of Medical Sciences and Technology, Jaipur, IND

**Keywords:** air leak test, gastrojejunostomy, hepaticojejunostomy, pancreaticojejunostomy, pancreatoduodenectomy

## Abstract

Background

Intraoperative air leak test (ALT) is routinely used to check anastomosis of the stomach, small intestine, and rectum. Its advantage is that it repairs the leak at the same time, thus preventing postoperative complications and re-exploration.

Method

ALT was done by insufflating the stomach with air to check the integrity of gastrojejunal anastomoses (GJ); the same air was pushed into the afferent jejunal limb to distend it up to hepaticojejunostomy (HJ) and pancreaticojejunostomy (PJ), thus testing the leak at all three anastomoses. ALT was performed in 16 patients (group 1) and the results were compared with 52 patients (group 2) who underwent pancreatoduodenectomy (PD) without ALT, between August 2019 and December 2022.

Results

ALT revealed a GJ leak in two and an HJ leak in one out of 16 patients, all of which were repaired immediately. It did not reveal any PJ leak. No postoperative bile leak was seen in group 1, while postoperative bile leak was seen in five of 52 patients in group 2 on postoperative day (POD) 1. High drain fluid amylase in the left drain on POD 3 was present in 11 patients (69%) in group 1 and 30 patients (44%) in group 2. A right drain was removed two days earlier and a left drain one day earlier in group 1. Reintervention and 30-day mortality were higher in group 2. Hospital stay was shorter (7.4 versus 8.2 days) in group 1.

Conclusion

Intraoperative ALT identified an anastomotic leak from GJ and HJ but not PJ, facilitating its immediate repair, thus preventing serious postoperative complications following PD. Whether ALT has any role in the detection of a PJ leak needs further validation.

## Introduction

Pancreatoduodenectomy (PD) is a complex high-risk surgical procedure performed for malignant and benign tumors of the periampullary region (the pancreatic head, ampulla, distal bile duct, and duodenum). PD is associated with high postoperative complication rate ranging 40%-60% [1], major complications such as pancreatic leak or fistula (4%-30%), delayed gastric emptying (6%-50%), wound infection (21%-23%), pulmonary complications (14%-17%), intra-abdominal collections (10%-12%), postoperative hemorrhage (12%-14%), biliary fistula (6%-7%), and reoperation (4%-5%) [2-5]. The mortality of PD has decreased in the last 15-20 years to less than 5% in high-volume centers, due to refinement in surgical technique, better postoperative care, and advances in interventional radiology [6].

Pancreatic and biliary anastomotic leaks are frequent complications following PD. These complications, in turn, lead to a chain of complications mentioned earlier and frequently require reintervention or reoperation. Hence, the prevention of leaks from pancreaticojejunostomy (PJ) and hepaticojejunostomy (HJ) after PD is important. An intraoperative air leak test (ALT) can help detect a leak and repair it immediately during the same surgery, thus preventing postoperative complications and the need for reintervention or reoperation. In the literature, ALT has been reported in colorectal anastomosis, small bowel anastomosis, and HJ. We report the results of a single surgeon-based pilot study on the use of ALT in PD.

## Materials and methods

Methodology

This is a retrospective study in which PD was performed on 68 patients between August 2019 and December 2022 at a tertiary care university hospital in India. All the patients who underwent PD were included in the study. The patients were divided into two groups according to whether ALT was done or not. Sixteen patients who were operated between January 2022 and December 2022 underwent ALT (group 1), while 52 patients who were operated earlier between August 2019 and December 2021 did not have ALT (group 2). The data were collected from a prospectively maintained database in the department. All the patients who underwent PD for either benign or malignant conditions and were more than 18 years of age were included in the study. Patients who underwent triple bypass as palliation were excluded from the analysis. This study was conducted to assess the effectiveness of ALT in detecting intraoperative anastomotic leaks (PJ, HJ, and gastrojejunal anastomoses {GJ}) and compare it to the postoperative outcomes (postoperative pancreatic fistula {POPF}, bile leak, and anastomotic leak). The Institutional Ethics Committee for Biomedical and Health Research of Mahatma Gandhi Medical College and Hospital issued approval MGMC&H/IEC/JPR/2023/315.

Statistical analysis

Statistical analyses were performed using Microsoft Excel 2023 version 16.73 (Microsoft Corp., Redmond, WA) for Mac. Continuous data are expressed as mean ± SD. The comparison of continuous or categorical variables was performed with Student’s t-test or the chi-square (χ^2^) test (or Fisher’s exact test), respectively. A P value of ≤0.05 was considered significant.

Surgical technique

We performed pylorus-resecting PD (PRPD) in the majority of the cases (65); pylorus-preserving PD (PPPD) was done in three cases. After the kocherization of the duodenum, the pancreatic head was lifted, and a tunnel was created anterior to the portal vein; the stomach was divided just proximal to the pylorus (PRPD), the hepatoduodenal ligament was dissected, and the gastroduodenal artery was divided; common hepatic dust was divided just proximal to the cystic duct junction. Jejunum was divided using a 60 mm blue linear stapler (Johnson & Johnson, New Brunswick, NJ), and the proximal jejunal limb was passed to the supracolic compartment in a retrocolic position, i.e., through a window in the transverse mesocolon. PJ was done using a modified Blumgart’s technique using interrupted Prolene 3-0 transpancreatic sutures and duct to mucosa using polydioxanone (PDS) 5-0 sutures. The pancreatic duct was stented using an infant feeding tube (IFT). HJ was performed in a single layer, end to side, using continuous PDS 4-0 suture. GJ was done in a single layer, end to side, using continuous PDS 3-0 suture. In cases of PPPD, duodenojejunostomy (DJ) was performed in a similar manner.

**Figure 1:**
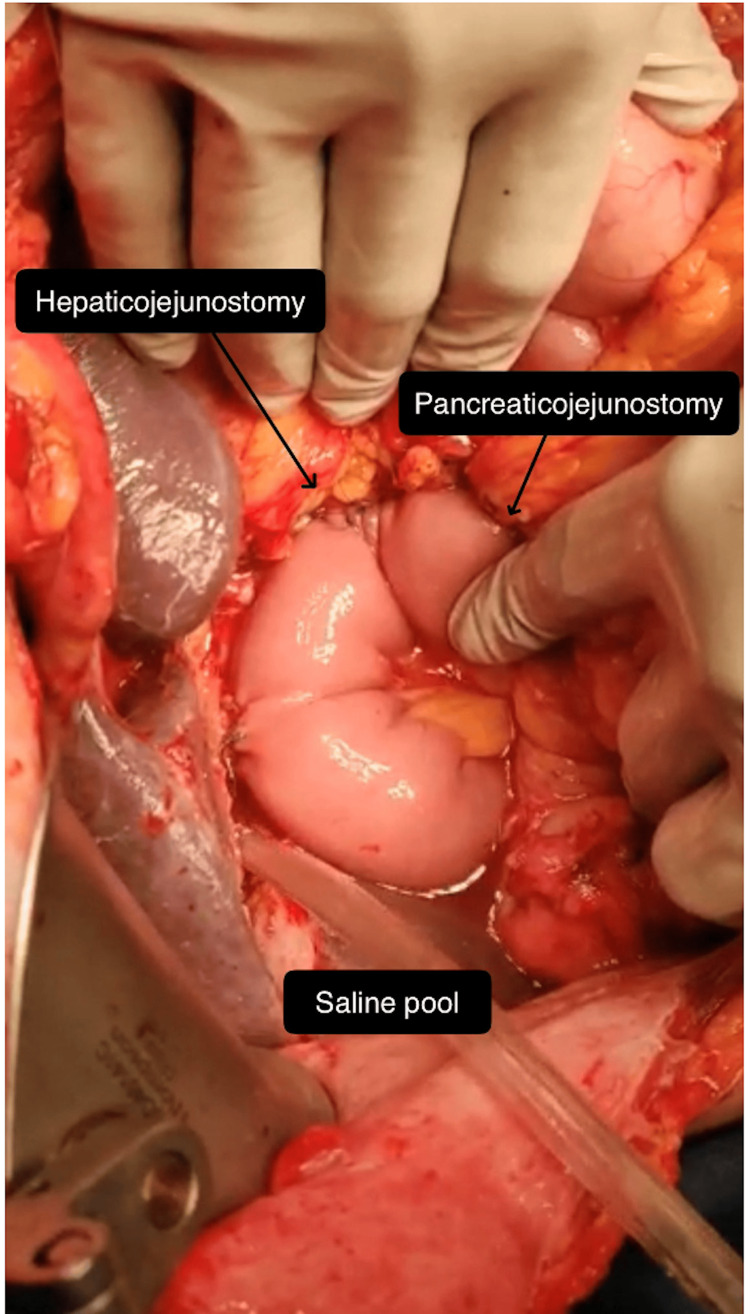
Intraoperative photograph showing air leak test with distended hepaticojejunostomy and pancreaticojejunostomy.

**Figure 2:**
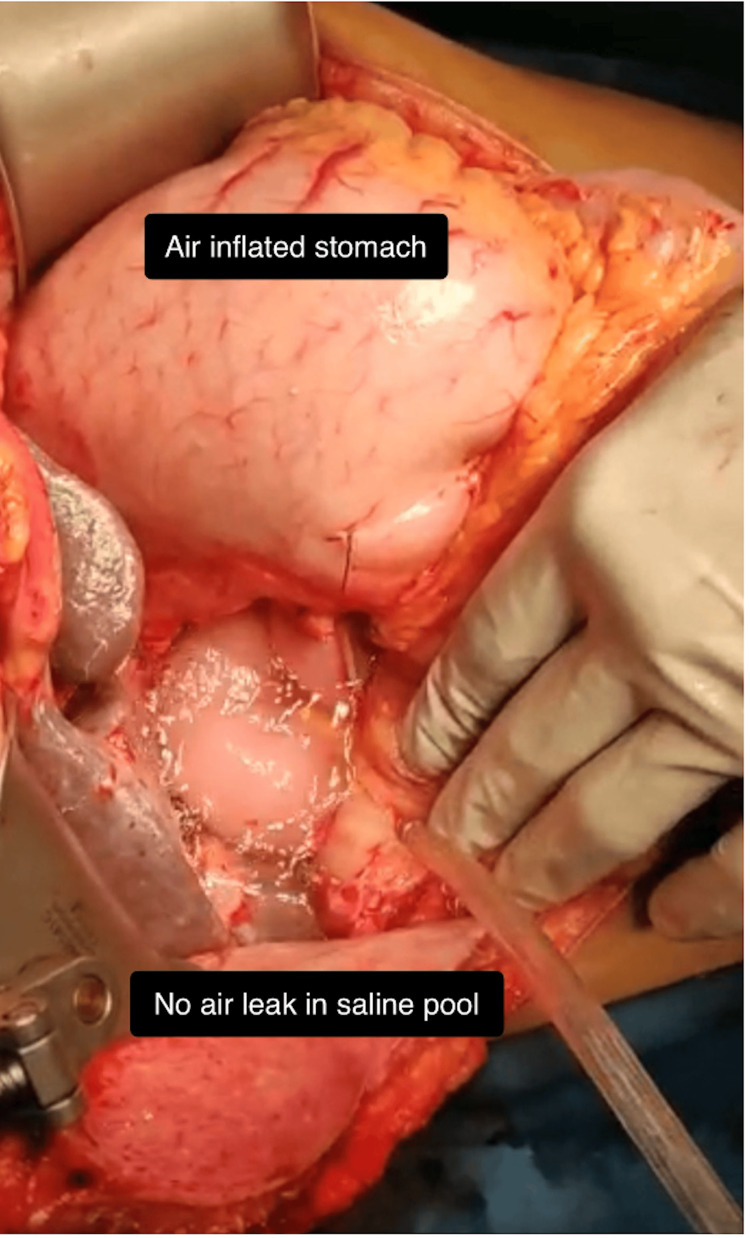
Intraoperative photograph showing air leak test with distended gastrojejunal anastomosis.

All patients underwent the same technique of PJ, HJ, and GJ/DJ after the resection of the specimen. After the completion of all three anastomoses, i.e., PJ, HJ, and GJ/DJ, a gauze test was done for HJ, and when no leak was found by a gauze test, then ALT was performed by insufflating the stomach with air (oxygen) via Ryle’s tube after the distal limb of the jejunum beyond the GJ was occluded with assistant’s fingers and filling the peri-anastomotic area (around the three anastomoses) with saline (Figures 1, 2). The rate of injected oxygen was kept at 4 L/minute. The pressure was not accurately quantified; however, it was clinically assessed by sufficiently distending the jejunum to make it tense (Figure 1 and Figure 2).

**Figure 3:**
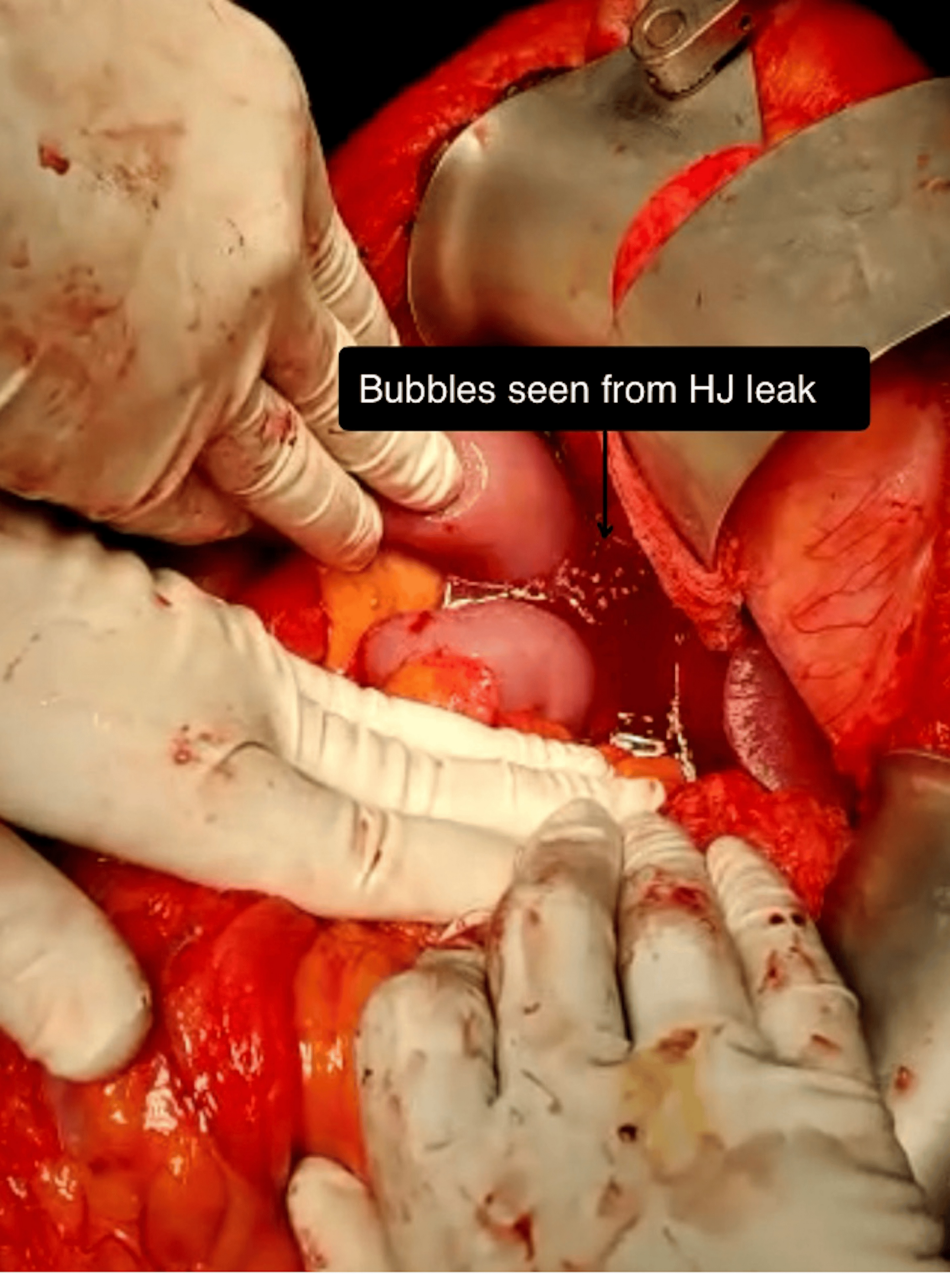
Intraoperative photograph showing air leak test with bubbles from hepaticojejunostomy (HJ), which was repaired immediately.

The GJ/DJ was first examined for any leak, as the stomach got distended. Air in the jejunum was then moved upward in the proximal jejunal limb to distend it up to the HJ and PJ sites, to test leaks at these anastomoses. If any air bubbles were seen coming from any anastomosis in the saline pool, then it confirmed a leak from that anastomosis (Figure 3).

As a protocol, two drains were routinely placed, one posterior and one anterior to the HJ and PJ. Prophylactic antibiotics were administered to all the patients for five postoperative days (POD). Octreotide was not given to any of the patients. An oral feed was initiated six hours after surgery with clear liquids, increasing to a soft diet over the next few days. The nasogastric tube was removed on postoperative day (POD) 1.

As a protocol, drain volume assessment was done daily, and drain fluid amylase levels were measured on POD 3 and 5 [7,8]. The drain was removed when the drain fluid amylase was less than three times the normal serum levels and/or the volume was less than 15 mL/day.

## Results

Both groups were comparable in demographic parameters (Table 1). Anastomotic leak was detected on ALT in three of the 16 patients in group 1 during surgery (P = 0.08). Out of these three leaks, a GJ leak was detected in two patients and an HJ leak in one patient. The site of the leak was repaired immediately using single-layer interrupted sutures during the surgery. The air leak test was repeated after the repair, and the absence of a leak was confirmed. A PJ leak was not found on ALT at surgery in any patient (Table 2).

**Table 1:** Demographic comparison of the two groups. Continuous (#) and categorical ($) variables were performed with Student’s t-test and the χ^2^ test, respectively. CBD, common bile duct; ALT, air leak test

Demographics	Group 1 (n = 16), ALT done	Group 2 (n = 52), ALT not done	P value	Statistical test
Age (years)^#^	55 ± 14	53 ± 11	0.26	Student’s t-test
Sex (male/female)^$^	10/6	37/15	0.27	Chi-square test
Site of lesion				
Periampullary	11	30		
Pancreatic head	3	10		
Distal CBD	0	7		
Duodenal	2	5		

**Table 2:** Comparison of intraoperative parameters. Continuous (#) variables were performed with Student’s t-test. ALT, air leak test; NA, not available

Parameter	Group 1 (n = 16), ALT done	Group 2 (n = 52), ALT not done	P value	Statistical test
Anastomotic leak detected on ALT^#^	3 (19%)	0	NA	
Texture of the pancreas^#^			0.38	Student’s t-test
Soft	12 (75%)	37 (71%)		
Firm	4 (25%)	15 (29%)		
Diameter of the pancreatic duct^#^			0.45	Student’s t-test
<3 mm	9 (56%)	29 (56%)		
>3 mm	7 (44%)	23 (44%)		
Diameter of the common bile duct^#^			0.09	Student’s t-test
<10 mm	4 (25%)	9 (17%)		
>10 mm	12 (75%)	43 (83%)		
Blood loss (mL)^#^	232 + 140	357 + 404	0.03	Student’s t-test
Duration of procedure (minutes)^#^	255 + 115	266 + 111	0.38	Student’s t-test

Comparing the outcomes of the two groups in the postoperative period, bile leak was not observed in any of the patients in group 1, while it was observed in five (10%) of 52 patients in group 2 on POD 1 (P = 0.01), which was found to be statistically significant. Only one patient required intervention with percutaneous catheter drainage (PCD) for bile leak on POD 3 in group 2 (P = 0.23). The rest of the patients had developed a controlled external biliary fistula, which was managed conservatively and stopped in due course of time. Drains could be removed earlier in group 1. Another patient in group 2 required PCD insertion for PJ leak, which was categorized as postoperative pancreatic fistula (POPF) grade B and persisted for a year. No postoperative intervention or reoperation was required for patients in group 1. Reintervention, reoperation, and 30-day mortality were more frequent in group 2 (Table 3).

**Table 3:** Comparison of immediate postoperative parameters. Continuous (#) and categorical ($) variables were performed with Student’s t-test and the χ^2^ test, respectively. ALT, air leak test; POD, postoperative day; GJ, gastrojejunal anastomoses; DJ, duodenojejunostomy; HJ, hepaticojejunostomy

Parameter	Group 1 (n = 16), ALT done	Group 2 (n = 52), ALT not done	P value	Statistical test
Intraoperative anastomotic leak detected on ALT	3 (19%) (GJ leak, two; HJ leak, one)	Not tested		
Bile leak on POD 1^$^	0	5 (10%)	0.01	Chi-square test
GJ/DJ leak	0	0		
Right drain fluid amylase (POD 3 > 150 U/L)^#^	10 (62%)	27 (52%)	0.41	Student’s t-test
Left drain fluid amylase (POD 3 > 150 U/L)^#^	11 (69%)	31 (60%)	0.18	Student’s t-test
Right drain fluid amylase (POD 5 > 150 U/L)^#^	2 (12.5%)	3 (5.7%)	0.21	Student’s t-test
Left drain fluid amylase (POD5 > 150 U/L)^#^	1 (6.2%)	4 (7.6%)	0.15	Student’s t-test
Right drain removal (days)^$^	8.0 + 7.5	10.5 + 9.5	0.38	Chi-square test
Left drain removal (days)^$^	11.0 + 9.7	12.7 + 11.7	0.16	Chi-square test
Delayed gastric emptying^#^	4 (25%)	10 (20%)	0.27	Student’s t-test
Reintervention^$^	0	2 (4%)	0.23	Chi-square test
Reoperation^$^	0	3 (6%)	0.31	Chi-square test
30-day mortality^$^	0	4 (8%)	0.34	Chi-square test
Postoperative hospital stay (days)^#^	7.4 + 3.3	8.2 + 3.6	0.25	Student’s t-test

High drain fluid amylase in the left drain on POD 3 was present in 11 patients (69%) in group 1 and 30 patients (44%) in group 2. The right drain was removed two days (eight versus 10.5) earlier and the left drain one day (11 versus 12.7) earlier in group 1 as compared to group 2. Hospital stay was shorter (7.4 versus 8.2 days) in group 1 as compared to group 2.

Three patients in group 2 required reoperation, one each for portal vein thrombosis, superior mesenteric vein thrombosis, and colocolic anastomotic leak in the postoperative period. The patient with portal vein thrombosis was managed with a polytetrafluoroethylene (PTFE) graft and had a long postoperative hospital stay; the other two patients could not be salvaged. There were two more deaths in group 2; one patient developed acute pulmonary embolism on POD 6, and the other patient got infected with the COVID-19 virus on POD 9, and both died due to respiratory complications.

## Discussion

An air leak test (ALT) is a simple test that can be done quickly (in a few minutes) and can confirm the absence of a leak of an anastomosis. The integrity of an anastomosis can be impacted by a variety of reasons, including inadequate nutrition, severe inflammation, and intestinal edema [9,10]. However, the ALT can only evaluate the mechanical component or the failure of the surgical technique. ALT has been used in various anastomoses. A randomized controlled trial (RCT) to detect anastomotic leaks in colonic anastomoses showed less leaks in the group of patients who had undergone ALT (n = 284/2395) [11]. This is similar to the results in our study (with a leak rate of 0% in group 1 and 9.6% in group 2). Intraoperative ALT done in multiple studies has detected leaks in 1.5%-24.7% of cases of colonic anastomosis by different authors [12].

In the present study, ALT was done to check leaks in all three anastomoses (PJ, HJ, and GJ/DJ) after PD. When a leak was detected at the GJ and HJ sites, intraoperative measures were taken to control the leak. In group 2 where ALT was not done, these leaks were not detected or corrected intraoperatively, thus leading to postoperative complications and the prolongation of hospital stay as compared to group 1. We did not detect any leak in the PJ in group 1 by the ALT. Postoperative drain fluid amylase was high in both group 1 and group 2. We, therefore, feel that ALT is not effective in detecting PJ leaks. In addition, there are several other factors that play a role in postoperative PJ leak, as many patients had increased drain fluid amylase, thus confirming PJ leak even in the absence of a leak detected by ALT.

Only one study from China has been published on ALT for PJ [13]. In this study, the air was insufflated via a trans-anastomotic catheter placed in the PJ to check only the PJ. Out of 46 patients tested with the insufflation test, 10 patients were found to have a leak from the PJ, which was reinforced with an interrupted suture. Out of these 10 patients, two patients showed grade A pancreatic fistula in the postoperative period. Pancreatic leak is the single most important factor responsible for the morbidity and mortality associated with PD [14]. Our study revealed that among these patients in whom ALT was performed for all three anastomoses, postoperative bile leak and pancreatic leak were observed less frequently, and patients suffered less morbidity and could be discharged early. The surgical strategy of ALT exhibited a promising outcome in terms of postoperative leak rates, drain removal time, and hospital stay. There are no articles published on air leak tests for PJ.

HJ leak is uncommon after PD, accounting for 6% of all major complications [15]. Only a handful of articles have been published on the HJ leak test [16]. In an Indian study, 63 patients underwent the air insufflation test for HJ. Air leaks during surgery were found in five patients and were secured on the operating table. Later, 2/5 patients exhibited bile leak; however, none of the patients tested negative for ALT and developed any postoperative bile leak [16]. ALT, however, might not be useful in detecting leaks from any aberrant bile duct.

Our study has several limitations. Firstly, it is a retrospective study in which inherent bias cannot be excluded. Secondly, the sample size is relatively small. Lastly, we compared it with the retrospective cohort, which was done in two different time frames. Larger prospective studies and RCTs with ALT are needed to examine the mechanical integrity of the anastomoses in PD, which can reduce postoperative morbidity and ultimately shorten hospital stays and lower expenses.

## Conclusions

Intraoperative ALT identified an anastomotic leak from GJ and HJ but not PJ, facilitating its immediate repair, thus preventing serious postoperative complications following PD. Whether ALT has any role in the detection of a PJ leak needs further validation.
